# Analysis of the Beliefs About Critical Competence in a Sample of Psychosocial and Socio-Educational Intervention Professionals in Master’s Degree Training

**DOI:** 10.3390/jintelligence13030039

**Published:** 2025-03-18

**Authors:** Francisco Jose Garcia-Moro, Diego Gomez-Baya

**Affiliations:** Department of Social, Developmental and Educational Psychology, Universidad de Huelva, 21007 Huelva, Spain; fjose.garcia@dpsi.uhu.es

**Keywords:** critical thinking, ethics, case study, social education, higher education

## Abstract

Critical thinking is a skill of great importance in our current and future society. Its value goes beyond all theoretical doubt although it requires more practical development, especially in terms of coordinated and evidence-based approaches. In addition, the ethical foundation must permeate the entire critical process, indicating what to criticize, for what, why, how, and when, elements that should not be left to improvisation or what is traditionally done. The aim of this research was to describe the ethical connotations that come together in the critical process. To this end, we focused on a group case study of undergraduate and graduate students of Psychosocial and Socio-educational studies in Spain, collecting information with instruments built ad hoc. The results show little practical awareness of the weight of ethics in critical decisions, producing a change in orientation regarding educational training to improve decision-making based on critical thinking and ethics.

## 1. Introduction

Critical thinking (CT) is an active metacognitive process that, through the integration of diverse skills, dispositions, and knowledge, helps make an informed judgment oriented towards action and/or problem resolution (Morancho and Mantilla 2020). CT may be a needed requirement to adequately deal with challenges in our globalized, contradictory, and hyper-informed society (Morin 2004; Wolton 2010; UNESCO 2010, 2015, 2019, 2021a, 2021b), with a complexity that leads to a feeling of vertigo and demanding new skills development (Saiz and Silvia 2023). Sánchez et al. (2019) proposed the following conceptions to define CT: (a) the ability to express a personal position; (b) the ability to express a founded point of view; (c) making a judgment; (d) a thinking ability that is part of a more complex cognitive process; and (e) reflection based on knowledge, analysis, understanding, and the processing of information. However, in the context of psychosocial intervention, the proposal of Bezanilla-Albisua et al. (2018) may be a more comprehensive definition, indicating that CT is oriented towards understanding problems, resolving them, and evaluating alternatives for decision-making. In this sense, CT means understanding a reality aimed at solving problems.

In the present work, CT is referred to as a competence, because CT is expected to go beyond the mere mastery of the skills and inexorably linking the critical dimension with the need to act in the immediate context (Jiménez Pérez 2023). Furthermore, considering the primary objective of various university degrees that seek to promote positive change in individuals and society, such as social education, teaching, or social communication, it is not enough to simply criticize. Rather, it is essential to be critical from a commitment to well-being, seeking truth, and addressing fundamental issues that directly impact our existence, such as responsibility, tolerance, commitment, and the value of family (García et al. 2024; Walker and LeBoeuf 2022). Thus, CT, at least in education for social change, goes much further than the mastery of certain cognitive skills, requiring social competencies that enable the solution of problems in a concrete and real space-time context (Halpern 1998). It is not enough to acknowledge its importance; one must also have the ability to put it into practice. (Sánchez et al. 2019).

From different national and international frameworks, CT training is a demanding social urgency (UNESCO 1998, 2009; World Economic Forum 2020). Also, in education, different types of companies and the labor market give it increasing importance (Dwyer 2023; Halpern and Dunn 2021; Saiz and Silvia 2023). These recommendations are considered in formal teaching, materializing in the different institutional ideologies. In this sense, the university context is ideal for promoting CT, with the teacher being a key element in promoting its development (Bezanilla-Albisua et al. 2018; Tamayo et al. 2015). However, because this competence requires one to respond to social demands and not only as an isolated training action disconnected from social reality, some educational practices show a lack of correspondence between what is considered necessary and what is actually realized in practice (Febres et al. 2017; Pithers and Soden 2000).

There are many authors from different sciences who have approached the concept of CT. Thus, there seem to be as many definitions appearing as authors. This issue reflects, on the one hand, a growing interest in research on the subject and, on the other, the disparity of proposals that appear when defining it (Ennis 1987; Lipman 2006; Facione 1990, among many others). From our point of view, it seems reasonable to think that talking about criticism in fields not linked to psychosocial intervention is not the same as those from different perspectives. In this sense, responsibility towards social change does not seem qualitatively similar when one wants to identify errors in discourse to when one plans to propose factual alternatives in order to promote the promotion of positive values in our society.

There is no doubt that our current society needs people who understand reality from a competency and narrative approach embedded in one’s own life story together with others in society (García et al. 2024). This call for civic and social responsibility is made from different levels, higher education being one of them. Training for training’s sake is no longer enough, but rather, its realization of integrating the socio-community reality appears as the basis that justifies its existence. Developing social policies that promote the well-being of people in society is not developing ideas, but an action based on those ideas requires an understanding of what to improve, how to achieve it, and why it is necessary to carry it out (Saiz and Silvia 2023). The university must be genuinely aware of this necessity beyond merely considering it an important aspect of higher education. It is of little use to advocate for its importance if those responsible for teaching this critical competence primarily understand it as a process of analysis and reasoning and scarcely as an element of action and commitment (Bezanilla-Albisua et al. 2018). Critical competence should be integrated into higher education teaching, from a competence perspective (Jiménez Pérez 2023) and with ethical reflection. Critical educational intervention requires reflecting on ethics to act in social situations (Pantoja 2012).

CT, understood as the ability to discern the essential from the residual and anecdotal aspects of our society (Facione 1990; Nieto et al. 2009; UNESCO 2021a, 2021b), is a crucial element highly valued by university students in psychosocial and educational programs (García et al. 2021). Considering that such thinking may be influenced by the field of study (Altuve 2010) and acknowledging that it comprises skills, dispositions, and knowledge (Lai 2011), we aim to describe the beliefs of a group of undergraduate and graduate students engaged in psychosocial and educational intervention studies regarding critical thinking. This analysis will cover their understanding of critical thinking, the importance they attribute to it, and the value they place on the university’s role in its development. Additionally, we are interested in exploring the value they assign to ethics within the CT process. This study objective arises from the conclusions in the work of Arum and Roksa (2011), who considered that university students in their first years do not have sufficient tools or maturity to learn critical competence and that this competence improves with age (Huber and Kunce 2016), although the students do recognize its usefulness (García et al. 2021, 2024).

For this research, we expected to understand CT beliefs as the set of knowledge, values, and ideas that people use to guide their lives and actions with a strong internal consistency (Pérez and Méndez 1995). In line with this, research on this topic has indicated that people do not use scientific theories when solving practical problems (Rodrigo and Correa 1999) but rather a set of less consistent beliefs that contingently influence the actions of an individual.

## 2. Materials and Methods

### 2.1. Data Collection and Sample Composition

In order to analyze the proposed research topic, we focused on the description of a group of undergraduate and graduate students from degree programs related to psychosocial and socio-educational intervention in Spain. Specifically, a non-probabilistic intentional sample of 100 subjects was utilized, comprising 50 men and 50 women, aged between 18 and 35 years (M = 24.5; SD = 3.41), who were enrolled in undergraduate and graduate studies related to the aforementioned degrees at the University of Huelva, Spain. In detail, 43 respondents were pursuing undergraduate degrees, while 57 were enrolled in graduate programs.

The design used is mixed with a descriptive–correlational part and a qualitative content analysis part insofar as it has been considered the option that can best dissect and describe the beliefs of the individuals subject to intervention with more depth and richness. Along these lines, a self-developed questionnaire has been used as an information-collecting instrument to learn the beliefs held by the investigated population. In turn, this information has been complemented and expanded with two focus groups, in which the knowledge of opinions and beliefs about critical competence and the importance of ethics as a regulatory element of criticism has been deepened. We specify this in more detail below.

### 2.2. Instrumentation

#### 2.2.1. Critical Competence Beliefs Questionnaire

In order to assess the beliefs and opinions of the subjects under investigation, a self-developed questionnaire was utilized, which has been employed in previous research (García et al. 2024). This questionnaire was validated through a panel of judges, achieving a reliability coefficient close to α = 0.69 (see Table 1). Items are described in Table 2. Given the complexity of the construct being analyzed, we consider this level of reliability acceptable for the present study, although it is important to note that, due to the descriptive nature focused on a group case study, it may not be as significant in the final results. We are interested in gaining an in-depth understanding of a group case; a study object–context that allows us to provide insights that contribute to a better understanding and improvement of the phenomenon under investigation (Stake 2005). For the analysis, the statistical package JASP 0.19.2 was utilized. 

The issues raised are detailed below:

**Table 2:** Critical competence beliefs questionnaire.

Questionnaire About Beliefs About Critical Competence in the Initial Training of Degrees Linked to Social Intervention
I consider that my level of knowledge of critical thinking is… I think the university favors critical thinking. I believe that today’s society places a lot of importance on critical thinking. The subjects taken at the university promote critical thinking. The current societal situation requires individuals with critical thinking skills. Critical thinking involves discerning the essential from the non-essential. I consider that critical thinking is essential for my life. I believe that knowledge is necessary to be a good critical thinker. Critical thinking is something you are born with. I think I have good critical competence. Empathy is necessary for critical thinking. Critical thinking requires greater effort for the person, which sometimes does not pay off. I have taken or am taking specific training courses in critical thinking. Having critical thinking makes you popular among others. Critical thinking can be defined as an attitude of critiquing everything with which one disagrees.

The following sociodemographic variables were added to these questions: sex, age, employment status, and studies (undergraduate–postgraduate).

The selected questions correspond to three fundamental dimensions that address the objective of our study, which can be grouped as follows: (a) the components that constitute CT, (b) the role of the university in the development of CT, and (c) self-perception regarding CT in oneself and in others (Table 3).

The first proposed dimension aligns with the description in the scientific literature of the elements that constitute critical thinking, such as skills like argumentation, discernment, and analysis; attitudes such as effort and motivation; and knowledge, which supports discourse (Facione 1990; Lai 2011; Nieto et al. 2009, among others). It also highlights the need to consider the weight of ethics within the equation (Facione 2007), using the concept of empathy as an inseparable element of ethics (Du et al. 2022; Kleinlogel and Dietz 2014).

The second dimension refers to the role of the university as an institution that should promote critical thinking (Bezanilla-Albisua et al. 2018) and to society’s demand for individuals trained in this capacity to address the various emerging challenges (UNESCO 2021a; World Economic Forum 2020).

The third dimension pertains to specific beliefs associated with critical thinking, aiming to explore opinions on its learnability, perceived difficulty, and self-assessment of possession, among others. This dimension allows for obtaining a complementary profile of the surveyed students’ beliefs.

#### 2.2.2. Focus Group

To complement this information, two focus groups were established. The first group comprised 10 participants (7 women and 3 men), while the second group consisted of 9 participants (6 men and 3 women). These participants were selected from the total sample used in the questionnaire, ensuring variability in terms of age, gender, undergraduate and postgraduate studies, and professional experience. Each focus group session lasted 40 min.

During these focus groups, various issues related to critical and ethical competence in the professional practice of psychosocial intervention were discussed (Table 4). The information was transcribed, and a procedure was implemented to identify units and propositions with significance to the proposed topic. These units and propositions were then coded and grouped according to thematic nuclei (Table 5), facilitating the construction of categories that enable content analysis.

When conducting the focus groups, Smithson’s (2000) recommendations were taken into account to ensure the reliability of the process. These included the preparation of the moderator as well as their knowledge of the subject matter and ensuring that information flowed equally and appropriately among all participants, avoiding situations where a few individuals dominated or influenced the opinions of others. To address this potential issue, participants were asked to write down their responses to general questions beforehand, and the discussion was initiated based on those responses.

### 2.3. Procedure

Regarding the procedure, the selection of the degrees and the academic years was carried out intentionally, administering the online self-report prepared ad hoc and used in part in previous research (García et al. 2024). The questionnaire took approximately 10 min to complete once written informed consent was obtained from all students. Subsequently, two focus groups were held with a selection of students who participated in the survey and who voluntarily wanted to take it. The session was recorded and transcribed to perform content analysis based on established categories. The duration of the focus groups was 40 min.

## 3. Results

### 3.1. Quantitative Study

#### 3.1.1. Descriptive Statistics and Non-Parametric Tests

Table 6 shows descriptive statistics. A total of 90% of the students surveyed believe that they have a high level of knowledge about CT, with 80% of men and 70% of women considering CT as essential and important for their lives. However, 42% of women are hesitant when considering whether they have critical competence—that is, to act critically—compared to 28% of men. In addition, 70% of the individuals indicate that they have not taken any training course on CT.

Regarding the role of the university in promoting such thinking, 44% of men say they agree compared to 76% of women, in this sense the difference being significantly positive in favor of the latter. However, 68% of both sexes believe that society does not support CT.

Focusing on the more structural aspects of CT, 68% of boys compared to 52% of girls consider that they know how to discriminate what is important from what is circumstantial in situations. Curiously, only 20% of men and 36% of women do not give importance to knowledge as one of the fundamental elements of good CT. In turn, regarding empathy as a dispositional component, approximately 60% of the subjects consider it important to develop good CT compared to 30% who consider it not necessary. Finally, practically 75% of the entire sample believes that developing CT involves a great effort for the person that sometimes does not pay off and, furthermore, does not make the person who acts like this popular (60% of the sample).

**Figure 1:**
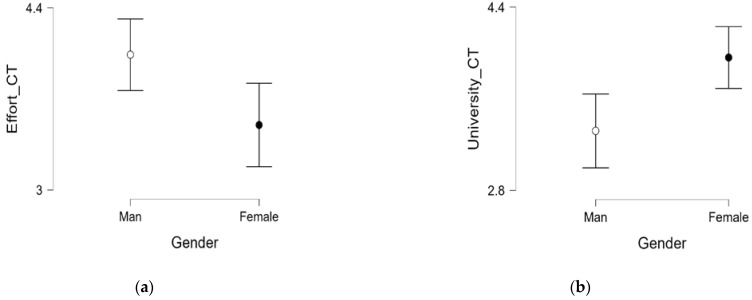
Non-parametric U Mann–Whitney (**a**) greater CT–sex effort; (**b**) the university CT–sex.

Regarding the variables “age” and “graduate–postgraduate”, no significant differences are obtained with any of the variables under study. Significant differences are obtained when we assess the differences between the sexes. Men believe that CT involves greater effort, while women believe that the university favors CT more than men do (Table 7 and Figure 1). Considering that when comparing samples we could not use parametric tests, since they do not comply with the principle of the homogeneity of the variances of the analyzed dependent variables, we used non-parametric tests to assess the possible differences between samples, with gender and work as dependent variables.

**Figure 2:**
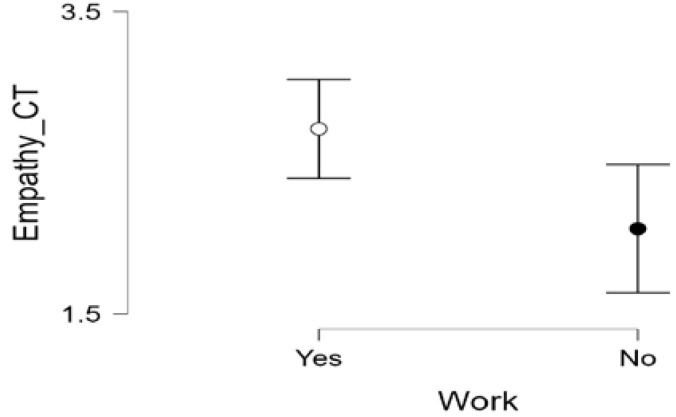
Non-parametric U Mann–Whitney empathy-being at work.

Finally, respondents who are working consider that empathy is a fundamental element of CT, unlike those who are not working (Table 8 and Figure 2).

#### 3.1.2. Correlations

Regarding the bivariate correlations, a significant correlation occurs at (*p* < .001), (*p* < 0.01) and (*p* < 0.05) between the following study variables: those who consider that they have a good level of knowledge of CT consider that they know how to discriminate the essential from the circumstantial (r = 0.397, *p* < 0.001); valuing CT as something essential for their own lives (r = 0.428, *p* < 0.001), although they also consider that it requires greater effort for people (r = 0.219, *p* = 0.028); and also not considering empathy to be a necessary element to develop critical thinking in their own professional role (r = −0.254, *p* = 0.011).

On the other hand, those who consider that the university favors CT believe that the current society gives a lot of importance to this thought (r = 0.300, *p* = 0.002), carrying out specific training courses on the topic (r = 0.235, *p* = 0.019), obtaining a very significant correlation between believing that one has a good knowledge of CT and having taken specific CT courses (r = 0.324, *p* = 0.001), and believing that one has good critical competence by having taken specific training courses on the topic (r = 0.338, *p* < 0.001).

We also highlight the correlation between the belief that critical thinking (CT) is innate and having high popularity (r = 0.329, *p* < 0.001), as well as the correlation between considering CT to be both innate and involving criticism of everything one disagrees with (r = 0.410, *p* < 0.001). This latter element shows a highly significant correlation with having high popularity (r = 0.325, *p* < 0.001).

Finally, a significant correlation is observed (r = 0.242, *p* = 0.015) between those who believe that knowledge is necessary for critical thinking and those who consider empathy to be equally essential. Table 9 shows bivariate correlations.

### 3.2. Qualitative Study

Below, we transcribe the results obtained in the focus groups, taking into account that the identification code that accompanies each statement is interpreted by the initials of male or female, age, working, graduate or postgraduate, and group.

To analyze this section, we follow the proposals of prominent researchers on critical thinking in defining its components (Ennis 1987; Epstein 2006; Facione 1990; Halpern 2003) in terms of skills and dispositions; however, we also consider motivational contributions (Valenzuela et al. 2014) and, of course, the component related to knowledge (Lai 2011).

We present the most relevant statements to be developed in the Discussion Section.

In response to the question of what critical thinking is, the discussion within the two focus groups can be summarized in the following statements:

- “It’s something that is talked about a lot nowadays, but we don’t really know it in depth.” (F34WP2).
- “… I don’t think we can afford to be cowards” (M20G2).
- “I don’t think it’s as easy as just wanting to do things. Even if you want to, you can’t always…” (F35WPG1).
- “…often it’s the pressure of the work environment that prevents you from doing something. I’ve had experiences in this sense of wanting to change something and not being able to do it because of the climate itself. It’s not always that easy” (M30WPG2).
- “For me, the automatism in which we are often immersed is what prevents us from acting critically” (M22G2).

The purpose of critical thinking:

- “… to improve the world” (M23G2);
- “to defend justice” (M23G1);
- “… to change the things we know are wrong or at least try” (M22WG1);
- “… injustices should not be allowed” (F22aG1);
- “… we have to be professional and improve society” (F22G1).

Components of critical thinking:

- Skills:
  ○ “knowing how to reflect and realize the errors that exist in society” (F20WG1).
- Dispositions:
  ○ Students participating in the focus groups consider that “CT is a fundamental attitude that all people should have in the face of the challenges of today’s society” (F21G2);
  ○ “it is a strategy that allows us to identify errors…” (M23G1);
  ○ “… to advance and improve our society by identifying problems…” (M22WG1);
  ○ “Strategy to change things…” (M26PG2);
  ○ “it is a way of facing problems that appear in life” (F31PG2).
- Knowledge:
  ○ “They are not just opinions as you have to go a step further. It is necessary to know what you think; a minimum knowledge since many things can be said and few of them be true…” (M28WPG2).
- Other elements:
  ○ “…In our way of understanding work, not everything goes; We must start from values, respect for the other person, with a highly developed empathetic attitude, with sincerity and knowing well what you can do and say depending on who you are with and at the moment” (F27WG1).

Characteristics of people who demonstrate critical thinking:

- Focused on cognitive aspects.
- Focused on attitudinal aspects:
  ○ “Having an opinion about something and knowing how to defend it” (M23G1);
  ○ “But it’s not enough to know things, you also have to defend them because there are people who know a lot and do nothing to change situations…” (M23G2);
  ○ “… improve the world” (M23G2).

- Focused on ethical aspects:
  ○ “…I also think it’s necessary to say that a good critical thinker seeks and defends the truth.” (F24G1);
  ○ “…I think that more than the truth, it would be what is convenient, what is necessary for the person with respect, without arrogance.” (F27WG1).

- Interaction of various dimensions:
  ○ “It is necessary to analyze, understand, know and also convince, otherwise what we do is useless…” (F29WPG1).

Role of ethics in critical thinking in professional performance:

- “…In our way of understanding work, not everything goes; we must start from values, respect for the other person, with a highly developed empathetic attitude, with sincerity and knowing well what you can do and say depending on who you are with and at the moment” (F27WG1).

## 4. Discussion

The training of future professionals in socio-educational and psychosocial intervention requires specific, rather than merely transversal, training programs in CT, following the recommendations of various competent institutions and organizations in the field (UNESCO 1998, 2009, 2010, 2015, 2019, 2021a, 2021b). The importance being attributed to CT is indisputable (Jiménez Pérez 2023; Saiz and Silvia 2023); in this regard, the sample aligns with this trend, giving a very high valuation to the importance of CT for their professional lives in socio-educational and psychosocial intervention, although it does not reach 100% of the sample.

However, knowledge about what CT entails and implies is significantly lower, and the responsibility for training in this area is practically anecdotal (Bejarano et al. 2014). Along these lines, Morancho et al. (2020) indicate that the lack of a coherent and shared conception of critical thinking (CT) in higher education has generated confusion regarding its development and assessment. There is great interest in CT while there is widespread ignorance about what it means or how to develop it in higher education. Similarly, studies such as that by Sánchez et al. (2019) reflect this paradoxical situation between aspirations and actions. While university students are aware of the importance of critical thinking, they continue to exhibit significant deficiencies in putting it into practice. There seems to be a notable lack of coherence between what is said and what is actually done—between intentions and actions.

Regarding whether respondents considered themselves to have adequate CT skills, no significant differences were observed between younger and older individuals. Furthermore, respondents value critical thinking as a necessary area for training; these results align with those obtained by Díaz-Larenas et al. (2019). However, there is generally little interest in pursuing such training, leaving attitude as a key component in the daily practice of critique.

Concerning gender differences, despite involving different research groups, there is a similarity with other studies (García et al. 2024). In both cases, women believe that universities foster CT more than men do; however, women also perceive that it requires less effort to implement compared to men. These results may be influenced by social upbringing and differentiated contextual development among participants (Escurra and Delgado 2008). Nevertheless, studies such as those by Albarracín-Vivo et al. (2024) or Sukarma (2019) indicate that female students demonstrate a greater propensity for critical thinking skills.

Arum and Roksa (2011) argue that first-year university students lack sufficient tools or maturity to develop critical competence; this skill improves with age (Huber and Kunce 2016), although its utility is widely recognized (García et al. 2021, 2024). Data indicated that the academic curriculum offered does not adequately develop CT. Notably, some studies have observed significant differences in CT between first- and third-year students; however, these differences may be mediated by contextual variables (Roohr et al. 2019). Other studies have even reported a decline in critical thinking throughout university education, with students losing both their skills and dispositions for critical thinking over time (de Oliveira Santos et al. 2025).

Although students initially struggle to define CT correctly, confusing it with mere opinions or attitudes of courage, they later manage to arrive at conclusions more aligned with research on the topic. That is, critical competence involves knowing how to argue, analyze, corroborate, and understand what appears before oneself (Ennis 1987; Facione 1990; Lai 2011; Paul and Elder 2003). Motivation is essential for this process (Valenzuela et al. 2014). It is necessary to know how to critique effectively (Morancho and Mantilla 2020). Critical thinking as a core competence seeks action to solve problems effectively and efficiently through reasoned argumentation (Rivas et al. 2022; Morancho et al. 2020), challenging individuals to evaluate situations rationally with the ultimate goal of making informed decisions and acting accordingly (García et al. 2021).

However, an ethical understanding is also necessary (García et al. 2024), as seen in the case of empathy—this being significantly more pronounced among working participants than non-working ones. Ethics is a central element for these individuals and even more so for future professionals in socio-educational and psychosocial intervention (Pantoja 2012). As Bezanilla-Albisua et al. (2018) point out, it is important to clarify the concept being addressed since “critical thinking” aimed at fostering analysis and organization differs from that which seeks social transformation through commitment. Critical thinking focuses not only on understanding problems but also on solving them through decision-making. It involves comprehending and evaluating problems within a community context but is always guided by ethical principles regulating professional conduct (Pantoja 2012).

Moreover, older working individuals exhibit a more complex view of critical competence compared to younger ones. This has been particularly significant in focus group discussions where participants highlight challenges; similar findings have been corroborated by other investigations (García et al. 2021; García et al. 2024). Ethics emerges as a fundamental element of critical thinking. Consistent with various studies, exposure to work environments or situations requiring effective decision-making fosters a greater development of critical thinking compared to contexts where such demands are absent or not explicitly addressed (Ejiogu et al. 2016; Ossa-Cornejo et al. 2020).

## 5. Limitations

The study presented here sheds light on the beliefs of future socio-educational intervention professionals in initial training regarding critical thinking and its real importance and the components involved in its development. However, it is necessary to keep in mind that these results cannot be generalized, since this was not a probability sample, but rather this research was based on a purposive sample, with the objective of describing beliefs about the critical thinking of specific groups. Nevertheless, we believe that this enriches scientific knowledge.

## 6. Conclusions

We find especially interesting the importance of the ethical component in critical competence in psychosocial and socio-educational intervention professionals, establishing an inseparable relationship between criticism for change and ethics that respects fundamental principles of respect This aspect means defending a transversality of certain components that define the critical work of these professionals while at the same time entails a review to integrate the ethical dimension, as a necessary element when understanding critical competence.

## Figures and Tables

**Table 1:** Frequentist scale reliability statistics.

			95% CI	
Coefficient	Estimate	Std. Error	Lower	Upper
Coefficient α	0.687	0.051	0.586	0.788

**Table 3:** Dimensions of the critical competence beliefs questionnaire.

Questionnaire About Beliefs About Critical Competence in the Initial Training of Degrees Linked to Social Intervention
The components that constitute critical thinking: I believe that knowledge is necessary to be a good critical thinker. Critical thinking involves discerning the essential from the non-essential. Empathy is necessary for critical thinking. Critical thinking requires greater effort on the part of the individual. Critical thinking can be defined as an attitude of critiquing everything with which one disagrees. The role of the university and society in the development of such thinking: I believe that the university promotes critical thinking. I think that contemporary society places great importance on critical thinking. The subjects studied at the university foster critical thinking. The current societal situation requires individuals with critical thinking skills. I have taken or am currently taking specific training courses in critical thinking. Self-perception regarding critical thinking in oneself and in others: I consider my level of knowledge about critical thinking to be… I believe I have good critical capacity. I consider critical thinking to be essential for my life. Critical thinking is something one is born with. Having critical thinking makes you popular among others.

**Table 4:** Focus group questions regarding critical competence beliefs.

Focus Group Questions Regarding Critical Competence Beliefs
If you had to give your future students an easily understandable definition of critical thinking, what would it be? What qualities or characteristics do you think a critical person has and what characteristics do you think do not define a critical person? Do you think that ethics should be a central element in a professional of psychosocial intervention? What relationship do you consider ethics should have when thinking and acting critically?

**Table 5:** Units of analysis for focus groups.

Units of Analysis for Focus Groups
Components of Critical Thinking ○ Skills ○ Dispositions ○ Knowledge ○ Other Elements Characteristics of People Who Manifest Critical Thinking ○ Focused on Cognitive Aspects ○ Focused on Attitudinal Aspects ○ Focused on Ethical Aspects ○ Interaction of Several Dimensions Role of Ethics ○ As a Transversal Element of Critical Thinking ○ As a Component of Critical Thinking ○ As Something That Is Sometimes Necessary and Sometimes Not ○ Other Beliefs

**Table 6:** Descriptive statistics.

Item	Variable Name	Mean	SD	Min.	Max.
I consider that my level of knowledge of critical thinking is…	Knowledge level	3.65	0.892	1	5
I think the university favors critical thinking.	University	3.64	1.087	1	5
I believe that today’s society places a lot of importance on critical thinking.	Society	2.3	0.893	1	5
The subjects taken at the university promote critical thinking.	Subjects	3.48	1.039	1	5
The situation of today’s society requires people with critical thinking.	Requirement	3.82	1.242	1	5
Critical thinking involves discerning the essential from the non-essential.	Discriminate	3.65	1.019	1	5
I consider that critical thinking is essential for my life.	Necessary	4.06	1.062	1	5
I believe that knowledge is necessary to be a good critical thinker.	Knowledge need	2.67	1.386	1	5
Critical thinking is something you are born with.	Innate	2.05	1.14	1	5
I think I have good critical competence.	Competence	3.49	0.948	1	5
Empathy is necessary for critical thinking.	Empathy	2.52	1.329	1	5
Critical thinking requires greater effort for the person, which sometimes does not pay off.	Effort	3.77	1.081	1	5
I have taken or am taking specific training courses in critical thinking.	Courses	2.15	1.282	1	5
Having critical thinking makes you popular among others.	Popular	2.34	1.085	1	5
Critical thinking can be defined as an attitude of critiquing everything with which one disagrees.	Criticizing	1.87	1.244	1	5

**Table 7:** Independent samples *t*-test of differences in the items concerning university and effort.

	IN	df	*p*
University	836.500		0.003
Effort	1596.000		0.012

Mann–Whitney U test.

**Table 8:** Independent samples *t*-test of differences in the item concerning empathy.

	IN	df	*p*
Empathy	1364.500		0.024

Mann–Whitney U test.

**Table 9:** Bivariate correlations.

	1	2	3	4	5	6	7	8	9	10	11	12	13	14
1. Knowledge level														
2. University	0.192 0.056													
3. Society	0.006 0.950	0.300 ** 0.002												
4. Subjects	0.227 * 0.023	0.789 *** <0.001	0.213 * 0.033											
5. Requirement	0.134 0.184	0.303 ** 0.002	0.095 0.349	0.302 ** 0.002										
6. Discriminate	0.397 *** <0.001	0.150 0.138	−0.028 0.784	0.122 0.226	0.157 0.118									
7. Necessary	0.428 *** <0.001	0.141 0.161	−0.083 0.411	0.193 0.054	0.299 ** 0.002	0.374 *** <0.001								
8. Knowledge need	0.118 0.242	0.155 0.124	0.171 0.090	0.251 * 0.012	0.135 0.179	−0.033 0.748	0.151 0.134							
9. Innate	−0.042 0.677	−0.010 0.923	0.144 0.154	0.048 0.637	0.042 0.678	−0.185 0.066	0.006 0.954	0.215 * 0.0032						
10. Competence	0.372 *** <0.001	−0.004 0.972	0.016 0.878	0.025 0.802	0.110 0.276	0.420 *** <0.001	0.572 *** <0.001	0.178 0.076	0.089 0.377					
11. Empathy	−0.254 * 0.011	0.117 0.247	0.250 * 0.012	0.117 0.245	0.002 0.983	−0.110 0.274	−0.058 0.566	0.242 * 0.015	0.196 0.051	−0.100 0.322				
12. Effort	0.219 * 0.028	0.023 0.817	−0.053 0.598	−0.072 0.479	−0.031 0.758	0.247 * 0.013	0.241 * 0.016	−0.044 0.661	−0.146 0.147	0.219 * 0.028	0.140 0.164			
13. Courses	0.250 * 0.012	0.235 * 0.019	0.146 0.149	0.233 * 0.019	0.322 ** 0.001	−0.029 0.775	0.179 0.075	0.324 ** 0.001	0.250 * 0.012	0.338 *** <0.001	0.102 0.313	0.142 0.160		
14. Popular	−0.085 0.403	0.062 0.540	0.311 ** 0.002	0.033 0.745	0.031 0.760	−0.129 0.201	0.508 0.997	0.022 0.831	0.329 *** <0.001	0.190 0.058	0.086 0.393	0.042 0.682	0.348 *** <0.001	
15. Criticizing	0.022 0.826	−0.125 0.217	0.199 * 0.047	−0.123 0.223	−0.041 0.682	−0.164 0.104	−0.078 0.440	0.186 0.064	0.410 *** <0.001	0.200 * 0.046	0.102 0.311	−0.037 0.711	0.310 ** 0.002	0.325 *** <0.001

* (*p* < 0.05), ** (*p* < 0.01), *** (*p* < .001).

## Data Availability

Data are available upon request to the first author.
